# Efficacy of monoclonal antibodies and maternal vaccination for prophylaxis of respiratory syncytial virus disease

**DOI:** 10.1038/s43856-025-00807-9

**Published:** 2025-04-16

**Authors:** Nele Plock, Jeffrey R. Sachs, Xiaowei Zang, Jos Lommerse, Kalpit A. Vora, Andrew W. Lee, S. Y. Amy Cheung, Brian M. Maas

**Affiliations:** 1 Certara, Radnor, PA USA; 2 Merck & Co., Inc., Rahway, NJ USA; 3 Present Address: Nalma, Oss, The Netherlands; 4 Present Address: Uniquity Bio, Malvern, PA USA

**Keywords:** Drug development, Paediatric research, Viral infection

## Abstract

**Background:**

Respiratory syncytial virus (RSV) is a leading cause of respiratory tract infection in infants and young children. The level of serum neutralizing antibodies (SNAs) is often used as a measure of protection against respiratory syncytial virus (RSV) infection.

**Methods:**

A qualified, model-based, meta-analysis efficacy prediction framework was used to understand the maternal vaccination-induced fold-increase in SNA titers necessary to achieve, over several study observation periods and study populations, similar protection to that of the monoclonal antibody clesrovimab (MK-1654).

**Results:**

Simulations indicated that 3-month and 6-month efficacy comparable to that predicted for passive immunization (clesrovimab) would require a maternal vaccine to increase SNA titers by 30- and 60-fold, respectively, higher than observed increases reported to date. Efficacy of maternal vaccination was predicted (for vaccines similar to those with published data) to be substantially lower for preterm infants compared to full-term infants, and substantially less over 6 months than over 3 months. Efficacy of passive immunization was predicted to be similar or higher in preterm infants than full-term infants and was similar for 3- and 6-month observation periods.

**Conclusions:**

Modeling can be used to reliably predict the efficacy of maternal vaccination for preventing RSV in infants. Passive immunization (e.g., with clesrovimab) is likely to provide more protection for preterm infants and for infants born outside the RSV season than that provided by current maternal vaccines. Maternal vaccination may provide partial protection from RSV disease to full-term infants born just prior to or during the RSV season.

## Plain language summary

Infection with respiratory syncytial virus (RSV) can lead to RSV disease, a respiratory illness which can lead to hospitalization in infants and young children. Two approaches for preventing RSV disease are immunization of the infant after birth and vaccination of pregnant people before delivery (maternal vaccination). We compared these two approaches with a computer model to predict which is more effective. Our results predict that immunization of both preterm and full-term infants could protect against RSV disease better than maternal vaccination. Maternal vaccination is predicted to partially protect full-term infants who are born just before, or during, a season when RSV is more common. Our results suggest immunization schedules (for infants) could potentially be timed with the seasons to protect even better against RSV disease.

## Abstract (web summary)

Plock et al. integrate clinical efficacy data on monoclonal antibodies and maternal vaccination for prevention of RSV disease in infants. Monoclonal antibodies are predicted to be more efficacious than maternal vaccination in preterm and full-term infants with maternal vaccination expected to partially protect full-term infants born just before, or during, an RSV season.

## Introduction

Respiratory syncytial virus (RSV) is a leading cause of respiratory tract infection in infants and young children^1^. It can cause severe illness, including bronchiolitis and pneumonia, and can lead to hospitalization, particularly in premature infants and individuals with underlying medical conditions^2^. RSV can also cause recurrent infection over the course of a person’s life, leading to long-term respiratory morbidity. Cautionary measures in response to the coronavirus disease 2019 (COVID-19) pandemic may have disrupted the typical seasonality of other respiratory viruses^3,4^. RSV seasons, for example, are emerging at unexpected times and with increased intensity^5,6^. This highlights an urgent need to protect vulnerable infants by developing highly effective RSV prophylactic agents.

The level of serum neutralizing antibodies (SNAs) is often used as a measure of protection against RSV infection^7^. Newborns with high concentrations of SNAs are less likely to have symptoms or complications from RSV infection than infants with lower concentrations^8^. A model-based meta-analysis (MBMA) demonstrated that SNAs represent an important correlate of protection for prevention of RSV disease across different populations, including the infant population^9^.

Passive immunization with monoclonal antibodies (mAbs) has proven to be beneficial in providing protection against RSV^10^. Monoclonal antibodies are highly specific, potent molecules that can target and neutralize RSV. Evidence from several clinical trials showed that mAbs protect against RSV disease in infants. In a randomized controlled trial, prophylactic administration of palivizumab, a humanized mAb, reduced the incidence of RSV hospitalization by 55% in preterm infants^11^. The recombinant mAb nirsevimab showed >75% efficacy against RSV-associated medically attended lower respiratory tract infection (MALRI) in late-preterm and full-term infants^12^. Additionally, a randomized controlled trial in high-risk infants showed that using motavizumab, another humanized mAb, as prophylaxis was 26% more effective than palivizumab in reducing the RSV hospitalization rate^13^. Extended–half-life mAbs could offer more predictable and potentially long-lasting protection^14–16^.

Maternal vaccination (of or relating to a pregnant person) against RSV is another approach that could protect infants indirectly. It provides passive immunity to a newborn via the usual transplacental transfer of maternal antibodies. These transferred antibodies can reduce the incidence and severity of RSV infection in infants, particularly in the first few months of life when they are at highest risk of infection^17^.

Results of several studies showed the efficacy of maternal vaccination in reducing the incidence of RSV disease in infants. In a randomized controlled trial performed across various geographical regions, maternal vaccination reduced the incidence of RSV disease in infants during the first 90 days of life, although efficacy varied by geographic location, and the primary endpoint was not met^18^. Results of another phase 3 trial of a recently approved bivalent RSV vaccine candidate showed a substantial reduction in the incidence of severe RSV-MALRI in infants through the first 6 months of life; protection was less over 6 months than over 3 months^19^. Literature is limited^18^ on how long transferred vaccine-induced antibodies last after birth and on factors that may affect the level of protection provided by the pregnant person^17^.

To our knowledge, maternal RSV vaccination has not been previously modeled based on a relationship between SNA titers and RSV incidence rate. Impact of maternal vaccination was evaluated using a compartmental mathematical model of RSV transmission^20^, utilizing the Susceptible–Exposed–Infectious–Recovered–Susceptible (SEIRS) model^21^. Although the seasonal pattern of RSV infection was described well in that model, the impact of maternal vaccination was based on assumptions, for example, around reduced susceptibility to infection at different times after birth. A model was published^22^ for a similar approach that assumed a boost of antibodies after maternal vaccination, with an associated assumed prolongation of protection. Other published results^17^ used simulations combining palivizumab competitive antibody (PCAs) exposure over time with a protective threshold PCA level^23^ determined using nonclinical experiments. Such a fixed target does not fully represent the expected dynamics of changing infection probabilities because of longitudinal changes in SNA titers. This will require a more integrated approach that combines predicted dynamic SNA titer data after maternal vaccination with previously acquired knowledge about the relationship between SNA titers and RSV disease^9^.

Clinical trial results from a direct comparison of maternal RSV vaccination efficacy with efficacy obtained via passive immunization are not yet available. Thus, the current study was designed to provide such a comparison by using an existing, qualified, model-based efficacy prediction framework^9^. The model was extended and qualified to enable efficacy predictions following maternal RSV vaccination. Using such a qualified model enables investigation of the immune response to maternal vaccination required to meet or exceed the protection provided by neonatal passive immunization, and to do so for a common endpoint.

Given data availability, clesrovimab (MK-1654), a novel RSV fusion (F) glycoprotein neutralizing mAb being developed to prevent RSV disease in infants, was used as a comparator here, and the modeling tools provided enable use of other comparators such as nirsevimab (which has similar neutralization)^24,25^. The simulation framework was used to provide a head-to-head prediction of the efficacy of maternal vaccination and passive immunization with a mAb for the prevention of RSV disease across infant subpopulations and for two study observation periods.

Simulations indicate that 3-month and 6-month efficacy comparable to that predicted for passive immunization (clesrovimab) would require a maternal vaccine to increase SNA titers by 30- and 60-fold, respectively, higher than observed increases reported to date. Efficacy of maternal vaccination is predicted (for vaccines similar to those with published data) to be substantially lower for preterm infants compared to full-term infants, and substantially less over 6 months than over 3 months. Efficacy of passive immunization is predicted to be similar or higher in preterm infants than full-term infants and is similar for 3- and 6-month observation periods.

## Methods

### Model description

#### MBMA model

Efficacy predictions for maternal vaccines and clesrovimab were based on virtual trial simulations using a published MBMA model^9^. Ethical approval (including study Institutional Review Board approval and practices for informed consent) and specific permission to use the published summary-level data was not required for this study as all data used were publicly available without restriction. No one involved in this study had access to any information that could identify the individuals from whom the data were originally derived.

In brief, the applied model was based on published clinical data from RSV vaccine and monoclonal antibody studies in infant, pediatric, adult, and older-adult populations to establish the relationship between time-varying SNA titers and RSV disease incidence rate at various levels of RSV disease severity. Given the high correlation between infant age and associated SNA titers, age does not need to be included as a predictor for risk of RSV disease in the MBMA model (in contrast with SEIRS models^20,21^). Instead, the model uses this relationship to predict the risk of RSV disease over a specified period given:

(1) The RSV incidence rate *R* by integrating the probability *p(t)* of contracting RSV at a particular day *t*, of the season:

$$R=100\cdot {\int }^{t={tlast}}_{\! \! \! \! \! t={tfirst}}p\left(t\right)\cdot {FOI}\left(t\right)\cdot {N}_{{norm}}\left(t\right)\cdot {dt}$$

Where *tfirst* and *tlast* represent the first and last day of the study, *FOI*(*t*) corresponds to an empirical RSV force of infection (FOI) weighting function, accounting for the likelihood and strength of natural RSV exposure present at a particular point during the year (FOI[*t*] was estimated from time courses of RSV rates in temperate regions in the United States from 2007 to 2012^26^, using a Gaussian function plus baseline^9^). *N*_*norm*_*(t)* describes the fraction of subjects present at day *t*, divided by the maximum number of subjects present at any time during the study.

(2) An underlying SNA titer time course profile used as input to a sigmoidal suppression function so that the probability of RSV infection (*p[t])* decreases with increasing SNA titers (i.e., risk of disease increases when titers, SN(*t*), decrease over time).

A detailed description of the full MBMA model is available in a previous publication^9^.

#### SNA titer models for maternal vaccination

Given the flexibility of the MBMA model to predict RSV incidence rate based on any SNA titer time course profile, the model was applied to predict RSV efficacy in infants after maternal vaccination. A prerequisite for these simulation-based predictions was the availability of SNA titer time courses from the start to the end of the scheduled observation period in the population of infants born to pregnant people vaccinated during pregnancy. Briefly, the SNA titer model is a piecewise model that naturally combines two functions: (1) a first-order SNA titer decay model applicable to infants born to vaccinated pregnant people and (2) a previously described natural baseline SNA titer model^9^. A commonly reported value in maternal vaccination studies is the GMR (also called fold increase) of SNA titers at birth of infants born to pregnant people vaccinated during pregnancy to SNA titers at birth of infants born to pregnant people who were not vaccinated before delivery of their neonate. The Supplementary Fig. S1 shows, and the Discussion section describes how this commonly reported GMR value can be used to provide (for a simulated population of infants) an infant’s SNA titer time course from birth onward as a function of gestational age (GA).

### Virtual trial population and study design

#### Model qualification in the context of maternal vaccination

The model described herein was qualified using maternal vaccine data from two phase 3 studies for which efficacy results were reported^18,19^. All studies used were conducted according to strict ethical standards, in accordance with the Declaration of Helsinki, applicable International Council for Harmonisation Good Clinical Practice guidelines as well as applicable laws and regulations. All trial participants provided informed consent from the mother or parent/guardian of each infant. The trials were undertaken at multiple sites and the protocol was reviewed and approved by regulatory authorities in all countries; and by ethical review committees for all sites. The vaccine studies used for validation were RSV F adj vaccine^18^ (previously in development) and RSVpreF^19^ (recently approved by the U.S Food and Drug Administration). For both studies, a virtual trial population was generated that matched the original study as closely as possible. Briefly, distributions of gestational ages were resampled from distributions derived from epidemiological natality data in the United States employing GA cutoffs as reported in the respective trial. Infant body weight over the duration of the trial was projected using appropriate sex distributions and standard curves to account for growth in full-term and preterm infants after birth. Further details on the methodology have been described elsewhere^9^. Details of the virtual population, simulated study design, and model parameters are presented in Table 1. All infants were enrolled at the time of birth. The trial simulations were based on a typical Northern hemisphere season with corresponding FOI. For both trials, efficacies were predicted and compared with reported results. The model was considered sufficiently qualified for maternal vaccination if confidence intervals between reported and predicted efficacies overlapped.

**Table 1:** Input parameters for maternal vaccine model qualification: virtual population, simulated study design, and model parameters

	RSV F adj vaccine	RSVpreF vaccine
Trial design		
Sample size (Infants)	2765:1430 (vaccine: placebo)	3500 per arm
Enrollment period	October–December	October–January
Simulated endpoint	RSV hospitalization	RSV MALRI and RSV hospitalization
Observation period	3 months	3 and 6 months
Virtual population		
Gestational age	≥37 weeks	≥24 weeks^a^
Sex	51.5% males	50% males
SNA titer at birth		
GMR_infant_^b^	2.175^c,^^18^	14.25 ^d,^^39^

GMR_infant_, geometric mean ratio for infants born to vaccinated pregnant people (divided by those born to unvaccinated pregnant people).

^a^Gestational age sampled from CDC natality distribution 2007–2017^45^.

^b^Calculated as the geometric mean average of reported RSV/A and RSV/B geometric mean fold ratio between infants born to vaccinated pregnant people compared with unvaccinated pregnant people, at time of birth.

^c^Using maternal ratios (vaccine:placebo) at time of delivery, assuming same titers in maternal participants and newborns (i.e., transplacental transfer ratio of 1).

^d^Values taken from 120-µg vaccine arm.

#### Maternal vaccination versus a single dose of clesrovimab

After model qualification for maternal vaccination, the model described herein was applied to understand the *GMR*_*infant*_ necessary to achieve similar efficacy to that predicted for the anticipated clesrovimab clinical dose. Predictions focused on the optimal target population for maternal vaccination, corresponding to infants born to pregnant people immediately before or during the RSV season. At birth, these infants were either protected through a hypothetical maternal vaccine or given the clinical clesrovimab dose or placebo. The overall study design and virtual population were identical to those in the trial for RSVpreF (for which *GMR*_*infant*_ is 14.25)^19^ as described in Table 1, with an observation period of 3 or 6 months. In the maternal vaccine arm, *GMR*_*infant*_ values of 15, 30, 45, and 60 (with appropriate variability) were evaluated to account for different *GMR*_*infant*_ values for various hypothetical maternal vaccines.

SNA titer time courses for the anticipated clesrovimab clinical dose were simulated using the model described previously^9^ and were used for efficacy predictions. Simulations were performed for the RSV-MALRI endpoint. Predicted efficacies are identical for RSV hospitalization, since efficacy parameters of the core suppression function in the underlying MBMA model did not support differentiation between the RSV-MALRI and RSV hospitalization endpoints. Results were assessed both for the mixed population of preterm and full-term infants, and for preterm and full-term infants separately. For the purposes of this analysis, preterm was defined as GA of <35 weeks.

### Simulations

For the purposes of simulation, the RSV season was assumed to correspond to a Northern hemisphere season^26^ (as described previously^9^). Using the population and design settings described in the Methods section, 1000 clinical trial simulations were run. For each simulation replicate, SNA titer time course profiles were predicted from birth onward for each infant enrolled in the study. For each time point of the study period, mean SNA titers were calculated using the individual titers from those participants enrolled in the study at this specific point in time. Mean SNA titers, weighted by the number of participants enrolled at a given time, were subsequently used to predict RSV incidence rates in each treatment arm. For each replicate, model parameters for the RSV MBMA were resampled from parameter uncertainty (with the same model applied across all study arms for a given simulation replicate), and interventions were randomly reassigned to individual participants.

Efficacy for each simulation replicate was calculated as the relative risk (RR) reduction between active and placebo arms. Mean predicted efficacies and 95% confidence interval values were calculated from parameter estimates obtained from fitting a normal distribution to the log (RR) values for all replicates. In addition, RR was calculated (using the same simulated dataset) between the maternal vaccination arms and the clesrovimab arm. The method of estimation of the mean predicted and confidence interval for this RR was identical to that used for efficacy. In this case, RR > 1 indicates that the use of (the simulated) maternal vaccination would be riskier (provide less protection) than use of clesrovimab. RR < 1 implies that use of clesrovimab would have a higher risk than maternal vaccination, and RR of 1 would imply that there is no predicted difference between the two arms. In the model qualification, an additional last step was performed to evaluate how well the predicted efficacies corresponded to efficacies reported for the respective maternal vaccines for the corresponding endpoint.

### Reporting summary

Further information on research design is available in the Nature Portfolio Reporting Summary linked to this article.

## Results

### Model qualification in the context of maternal vaccination

**Fig. 1:**
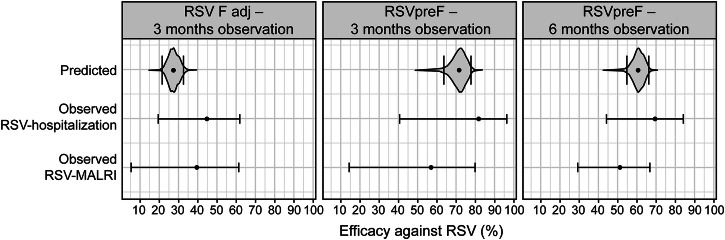
Consistency of predicted and reported maternal vaccine efficacies. For RSV F adj, the primary endpoints RSV-associated medically significant lower respiratory tract infection up to 90 days of life and RSV-associated lower respiratory tract infection with severe hypoxemia up to 90 days of life are used, respectively, for observed RSV MALRI and RSV hospitalization efficacies. For RSVpreF, the respective endpoints were medically attended lower respiratory tract illness and severe medically attended lower respiratory tract illness, with the assumption that severe illness corresponds to hospitalization. Data available (for the MBMA model) did not enable different predictions for different RSV endpoints, so that only a single prediction is available. Violins represent model-based efficacy distribution predictions, with black dots and vertical black lines corresponding to predicted median and 95% confidence interval (RSV F adj: 27.2% [21.6–32.5%] for 3 months of observation; RSVpreF: 71.8% [63.8–77.8%] and 60.9% [54.9–66.1%] for 3 and 6 months of observation, respectively). Black points and error bars represent observed point estimates of RSV efficacy along with 95% confidence interval in the respective treatment arm (observed data from Madhi et al.^18^ and Kampmann et al.^19^). RSV F adj, simulated *n* = 2765:1430 (vaccine:placebo); RSVpreF, simulated *n* = 3500:3500). MALRI medically attended lower respiratory infection, MBMA model-based meta-analysis, RSV F adj adjuvanted RSV fusion protein (F) vaccine, RSVpreF RSV F vaccine with prefusion conformation.

Alignment between predicted (simulated) and observed efficacy is shown in Fig. 1 (numerical values provided in Supplementary Table S1). Lower efficacy was predicted for RSV F adjuvant (RSV F adj) vaccine compared with RSV prefusion F (RSVpreF) vaccine, which is in line with the overall efficacies reported. Although the mean predicted efficacy of RSV F adj vaccine was lower than the point estimate reported, the small sample size resulted in wide confidence intervals, and there was a large overlap in confidence intervals for the efficacy endpoints. The predicted efficacy of RSVpreF vaccine for RSV MALRI and RSV hospitalization was contained between point estimates for these endpoints, and confidence intervals overlapped for all scenarios simulated. The model accurately predicted waning efficacy with longer observation periods. Based on the prespecified criteria, qualification of the model was considered sufficient for the model to be used in the context of maternal vaccination.

### Maternal vaccination versus clesrovimab treatment

**Fig. 2:**
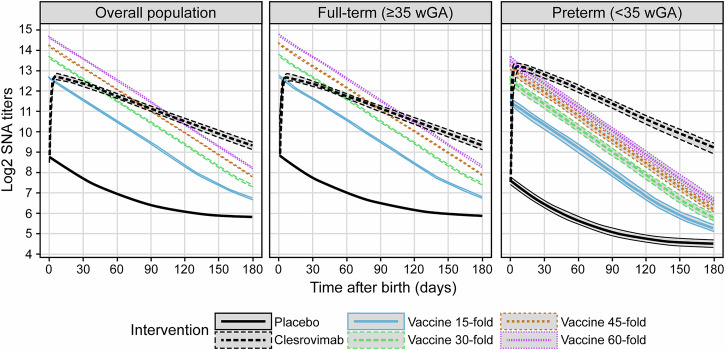
Predicted SNA titers from maternal vaccination and clesrovimab prophylaxis in the different populations. Predicted SNA titers at later time points are higher from passive immunization with clesrovimab than from maternal vaccination. At early time points, titers from passive immunization with clesrovimab are also higher if the maternal vaccination *GMR*_*infant*_ is <15-fold. The overall population (*N* = 3500 per arm) corresponds to the joint population of preterm (*N* = 150 per arm) and full-term (*N* = 3350 per arm) infants. Profiles shown here were created relative to time since birth. Lines and shaded areas represent median and 95% confidence interval of predicted mean log2 SNAs across simulation replicates. Details of simulations in text. *GMR*_*infant*_, geometric mean ratio for infants born to vaccinated pregnant people (divided by those born to unvaccinated pregnant people); SNA serum neutralizing antibody, wGA weeks of gestational age.

A comparison of simulated SNA titers for maternal vaccine and clesrovimab is presented in Fig. 2. Maternal vaccination and passive immunization with clesrovimab both resulted in high SNA titers, compared with placebo, at the time of birth. SNA titers were higher for the maternal vaccination arms at the geometric mean ratio for infants (*GMR*_*infant*_) chosen for simulation. However, SNA titers in infants born to pregnant people who had been maternally vaccinated decreased more rapidly (half-life of approximately 30 days^27–29^) than SNA titers in infants after passive immunization (half-life of approximately 45 days). As a result, SNA titers in the maternal vaccination arms decreased below those in the passive immunization arm as soon as infants were roughly 3 months old and were substantially lower from about 1 month after this point until the end of the period of interest.

It was established that, after maternal vaccination, SNA titers at birth would be lower in preterm infants than in full-term infants (Fig. 2) due to lower transplacental transfer of immunoglobulin G (IgG) in preterm infants^30,31^. In full-term infants, the maximum SNA titers between passive immunization with clesrovimab and maternal vaccination were similar with a *GMR*_*infant*_ value of ~15-fold. In contrast, *GMR*_*infant*_ values of ~45- to 60-fold were required for preterm infants. Conversely, clesrovimab at the anticipated clinical dose was predicted to yield slightly higher SNA titers in preterm infants than in the full-term infant population due to the lower birth weight in preterm infants.

Mean simulated SNA titers were used to predict RSV incidence rates in each treatment arm. The RR of each of the maternal vaccination arms was calculated relative to passive immunization with clesrovimab, and efficacies for the maternal vaccination arms and clesrovimab were calculated relative to placebo. The simulations account for the range of possible relationships between SNA titer and protection (and the variability in titers produced by the immunizations), and the RR calculation ensures that the protection provided by the two prophylactic agents are compared under the same estimate of that relationship. Because comparing the confidence intervals of efficacies is likely to compare the worst-case scenario for clesrovimab with the best-case scenario for the maternal vaccination (or *vice versa*), the RR results are a more appropriate basis of comparison of these two prophylactic approaches.

**Fig. 3:**
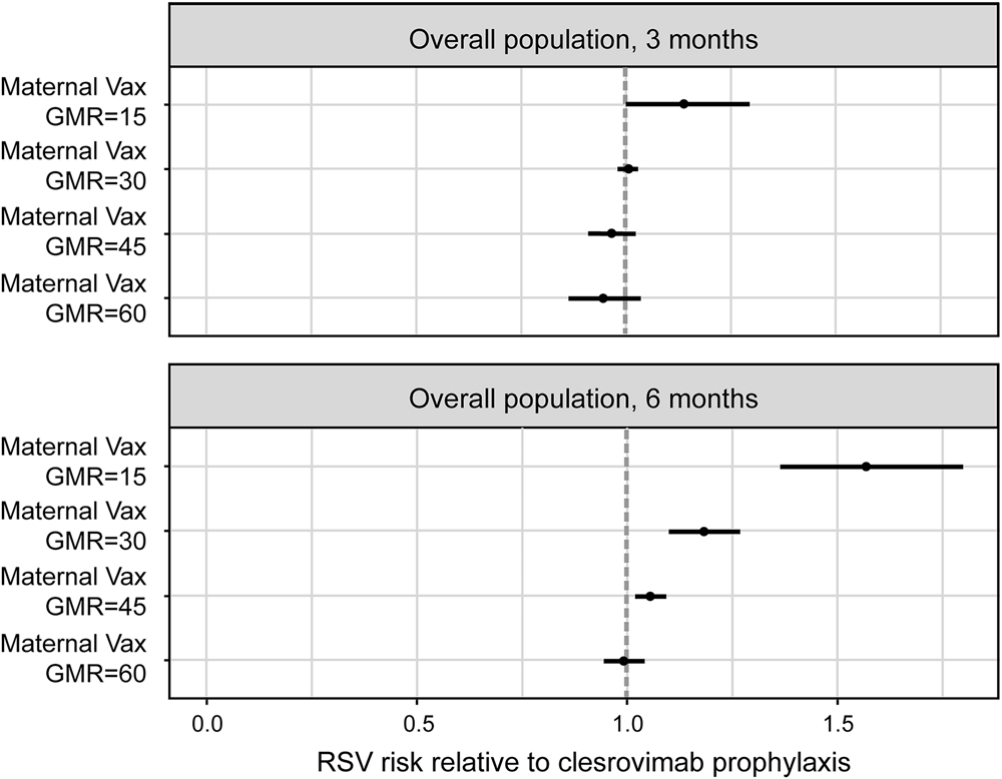
Predicted RSV risk with maternal vaccination relative to clesrovimab prophylaxis in the overall population. Clesrovimab full RSV season protection is projected to be superior to protection from maternal vaccines. Top and bottom rows show prediction results for observation periods of 3 and 6 months, respectively (*N* = 3500 per arm). Data are presented as risk relative to passive immunization with clesrovimab and its associated 95% confidence interval. RR > 1 (to the right of the dashed line) indicates better protection by clesrovimab and vice versa for RR < 1. Simulated RRs apply for all RSV endpoints: data available (for the MBMA model) did not enable different predictions for different RSV endpoints, so that only a single prediction is available. MBMA model-based meta-analysis, RR relative risk, RSV respiratory syncytial virus.

The predicted RRs are presented in Fig. 3 for the overall study population (i.e., preterm and full-term infants). Clesrovimab was predicted to provide greater protection over 6 months than all maternal vaccine arms except the one simulated with a 60-fold increase, which reached comparable protection to that of clesrovimab. For the shorter 3-month period, it was predicted that the 15-fold GMR would be less protective than passive immunization with clesrovimab, while simulated maternal vaccination arms that have at least a 30-fold GMR showed a predicted trend toward being as or more protective than clesrovimab.

Efficacy predictions for the overall study population are shown in Table 2 and Supplementary Fig. S2. The efficacy of clesrovimab was predicted to be 75.4% and 75.7% for observation periods of 3 and 6 months, respectively: in placebo-treated infants, titers 3 to 6 months after birth are lower than during the first 3 months, whereas clesrovimab titers are still high, making the relative difference (between clesrovimab and placebo) higher over the second 3-month period and, thus, nominally higher for the 6-month period. The increase in infant SNA titers at birth (induced via transplacental antibody transfer from vaccinated pregnant people) necessary to achieve protection similar to that of clesrovimab for 3 months was 30-fold; an increase of 60-fold was necessary to achieve similar protection for 6 months.

**Table 2:** Clesrovimab efficacy predicted to be more durable than that of hypothetical maternal vaccines

Intervention	Predicted 3-month efficacy % [95% CI]	Predicted 6-month efficacy % [95% CI]
Maternal vaccination (GMR_infant_ = 15)	72.1 [64.4–78.1]	61.8 [56.0–66.9]
Maternal vaccination (GMR_infant_ = 30)	75.3 [68.3–80.7]	71.1 [65.3–75.8]
Maternal vaccination (GMR_infant_ = 45)	76.3 [69.3–81.7]	74.4 [68.6–79.0]
Maternal vaccination (GMR_infant_ = 60)	76.8 [69.7–82.2]	76.0 [70.2–80.6]
Clesrovimab	75.4 [68.4–80.8]	75.7 [69.6–80.6]

Data are presented as mean predicted efficacy with associated 95% confidence interval. GMR_infant_, geometric mean ratio for infants born to vaccinated pregnant people (divided by those born to unvaccinated pregnant people).

Simulated efficacies apply for all RSV endpoints: data available (for the MBMA model) did not enable different predictions for different RSV (severity level) endpoints, so only one prediction is available across the endpoints. As explained in the text, overlap in the confidence intervals does not necessarily imply comparable protection, whereas the RR estimates provide a reliable basis for comparison. The efficacies are plotted for comparison in Supplementary Fig. S2.

**Fig. 4:**
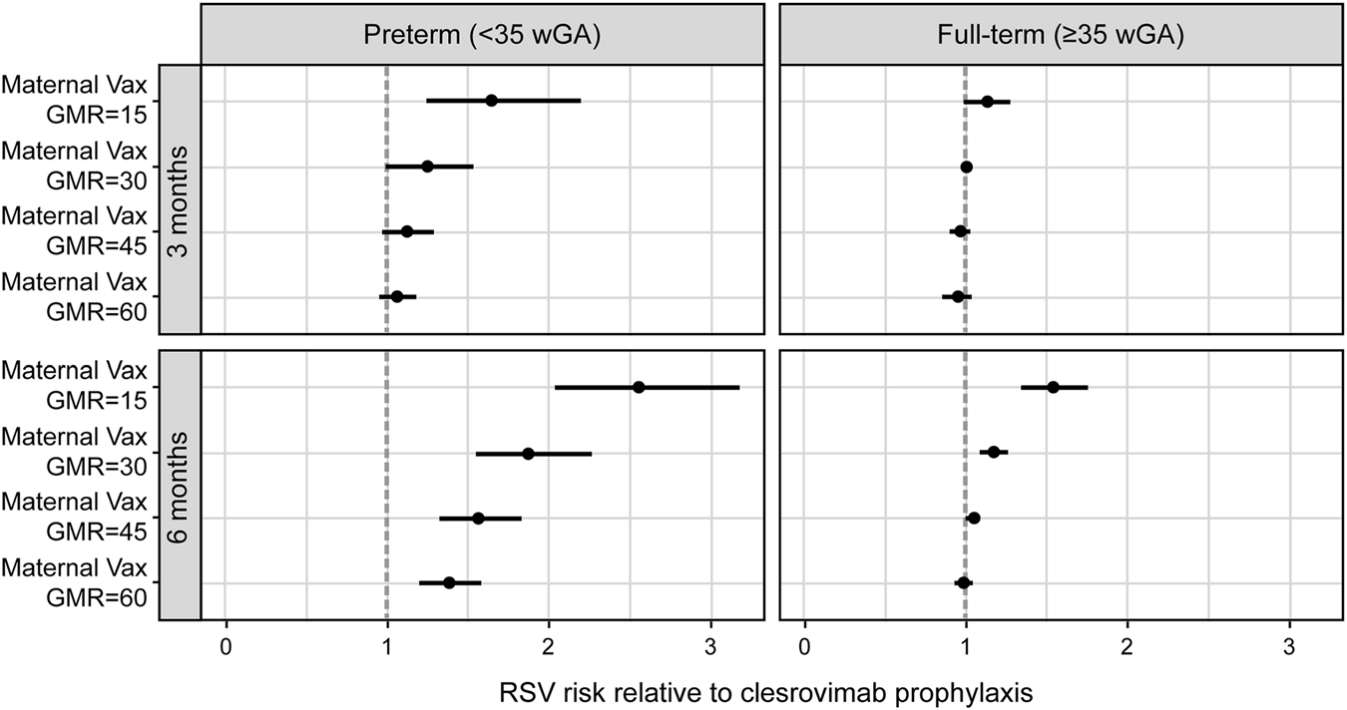
Predicted RSV risk with maternal vaccination relative to clesrovimab in preterm and full-term infants. Preterm: *N* = 150 per arm; full-term: *N* = 3350 per arm. Clesrovimab protection is projected to be more durable and similar or superior to protection from maternal vaccines. Top and bottom rows show predicted results for observation periods of 3 and 6 months, respectively. Data are presented as risk relative to passive immunization with clesrovimab and its associated 95% confidence interval. RR > 1 (to the right of the dashed line) indicates better protection by clesrovimab and vice versa for RR < 1. Simulated RRs apply for all RSV endpoints: data available (for the MBMA model) did not enable different predictions for different RSV endpoints, so that only a single prediction is available. MBMA model-based meta-analysis, RR relative risk, RSV respiratory syncytial virus, wGA weeks of gestational age.

The RRs and efficacies were also estimated separately for preterm and full-term infant subpopulations. The RR results are shown in Fig. 4, and efficacy results are presented in Table 3 and in Supplementary Fig. S3. Preterm infants, the majority of the population most at risk, have substantially better (lower) predicted risk for passive immunization with clesrovimab than with maternal vaccination (Fig. 4). The predicted RR ratio tended to be lower (smaller) for full-term infants; passive immunization still tends to be superior.

**Table 3:** Clesrovimab efficacy predicted to be more durable than that of hypothetical maternal vaccines, especially in the preterm population

Intervention	Predicted 3-month efficacy, % [95% CI]		Predicted 6-month efficacy, % [95% CI]	
	Full-term	Preterm	Full-term	Preterm
Maternal vaccination (GMR_infant_ 15)	71.9 [64.2–77.9]	67.7 [59.9–73.9]	62.2 [56.3–67.4]	46.9 [38.1–54.5]
Maternal vaccination (GMR_infant_ 30)	75.0 [67.9–80.5]	75.8 [69.6–80.7]	71.2 [65.4–76.0]	61.0 [53.9–67.1]
Maternal vaccination (GMR_infant_ 45)	76.0 [68.9–81.4]	78.1 [72.1–82.9]	74.4 [68.6–79.1]	67.6 [61.2–72.9]
Maternal vaccination (GMR_infant_ 60)	76.5 [69.3–82.0]	79.3 [73.1–84.0]	75.9 [70.0–80.7]	71.3 [65.4–76.2]
Clesrovimab	75.0 [67.8–80.5]	80.5 [74.2–85.3]	75.4 [69.2–80.4]	79.3 [74.0–83.6]

Data are presented as mean predicted efficacy with associated 95% confidence interval. GMR_infant_, geometric mean ratio for infants born to vaccinated pregnant people (divided by those born to unvaccinated pregnant people).

Predicted 3- and 6-month efficacy for clesrovimab and for hypothetical maternal vaccines in preterm and full-term study subpopulations. Full-term is defined as ≥35 weeks’ GA, preterm is defined as <35 weeks’ GA. Simulated efficacies apply for all RSV endpoints: data available (for the MBMA model) did not enable different predictions for different RSV (severity level) endpoints, so only one prediction is available across the different endpoints.

For the full-term populations (representing ~96% of the overall population), efficacy predicted for both immunization approaches was very similar to that of the overall population (Table 2). In addition, efficacy of maternal vaccination decreases with a longer observation period. The efficacies of preterm and full-term populations are also similar for the 3-month observation period, especially for *GMR*_*infant*_ ≥ 30 (Table 3). In preterm infants and with a shorter observation period of 3 months, predicted efficacy is similar to that of clesrovimab only for a *GMR*_*infant*_ ≥ 30.

For the 6-month observation period, the group of preterm neonates is notably different than the overall and full-term populations (Fig. 4 and Table 3). This period has substantial predicted differences between preterm and full-term neonates. In all maternal vaccination scenarios of preterm neonates with 6 months of observation, there is substantially worse (higher) RR in the maternal vaccination group. In the preterm population, 6-month efficacy is expected to decrease compared with that for 3 months, with efficacies lower than 50% predicted for a *GMR*_*infant*_ ≤ 15. For the longer observation period, maternal vaccination efficacies >70% are only predicted when *GMR*_*infant*_ is 60. Overall, maternal vaccination is predicted to be inferior to passive immunization over a 6-month period, except in full-term infants at the *GMR*_*infant*_ ≥ 45 (Fig. 4).

## Discussion

It is commonly understood that the most salient transfer of protective immune response from pregnant people to fetus is via transplacental transfer^32^. Use of SNA titers to predict protection in neonates is further supported by an existing qualified MBMA model^9^, which was developed using published clinical data from RSV vaccine and mAb studies in infant, pediatric, adult, and older-adult populations. This model showed that SNA titer time course profiles are predictive of protection from RSV, with similar protection for active and passive immunization given similar titers; that is, the same model, using only the SNA titer as a predictor, was equally predictive for exogenous mAbs and endogenous, polyclonal vaccine responses. Therefore, RSV efficacy after maternal vaccination is predictable given appropriate SNA titer time course profiles.

The current analysis assumed the SNA titer is a sufficient predictor of RSV efficacy regardless of the source of antibodies. After qualifying the titer-based predictive model for predicting efficacy of maternal vaccination, the work leveraged the approach to assess the immune response required of maternal vaccination to provide similar or greater protection than passive neonatal immunization. While clesrovimab was used here for comparison, the framework can be applied to any approach that increases neutralizing titers in neonates.

For maternal vaccination, it was assumed that the neonate SNA titers would decrease with the half-life derived using SNA titers (for the first 30 days from birth) for neonates born to unvaccinated pregnant people. Such a half-life estimate is well supported by data^33,34^ and literature^27,28^. In the absence of SNA titer boosts due to natural infection (assuming infants are protected from infection by the maternal vaccine when titers are high), this first-order rate of titer decay was preserved up to a time where SNA titers were approaching levels seen in infants born to unvaccinated pregnant people. At this point, it was assumed RSV infection will occur at a similar rate as in the control groups. As a consequence, from this time onwards the shape of the SNA titer profile corresponded to that described for infants born to unvaccinated pregnant people from birth onwards, with the average over the population increasing due to the small portion of infants with infection-boosted titers. Mathematically, this scientific understanding was described with a piecewise function, where the time point switching from one component function to the other was defined to provide continuity with the natural SNA titer levels at birth. Supplementary Fig. S1 shows the resulting natural shape of the resulting titer-versus-time profile.

When comparing SNA titers across simulated prophylaxes for preterm and full-term infants, a consistent pattern emerges. In maternal vaccine arms, preterm infants exhibit lower SNA titers than full-term infants at birth, whereas the opposite holds true for infants treated with clesrovimab. This aligns with the expectation that preterm infants will have lower titers at birth than full-term infants due to the incomplete transplacental IgG transfer in the preterm infants^30,31^. The consistent fold-increase approach using GMR values after maternal vaccination results in predicted titers in preterm infants being lower than those of full-term infants. Conversely, clesrovimab is administered at a fixed dose regardless of GA or body weight, leading to higher concentrations and SNA titers in preterm infants due to their lower body weight.

### Model qualification for maternal vaccination

Efficacy was predicted for two maternal vaccines to qualify the MBMA model in the context of maternal vaccination: RSV F adj (previously in development)^18^ and RSVpreF (recently approved by the U.S. Food and Drug Administration)^19^. The model captured the qualitative differences between the two vaccines. The efficacies of RSV F adj and RSVpreF were predicted to be 27.3% and over 70%, respectively, for a 3-month observation period.

Results were largely in agreement with reported efficacy^18,19^. Reported observed confidence intervals always included the predicted efficacy (reported observed point estimates were outside of predicted 95% confidence intervals). The model accurately predicted the expected^35,36^ decrease in efficacy as the observation period increased, an effect that was seen for both the maternal vaccines used in the qualification.

Variability in vaccine efficacy is observed across region/country, income status, and RSV subtype. In studies used for model qualification, factors such as medical practice may differ from those in studies included in the MBMA (e.g., in South Africa, which accounts for >~50% of participants enrolled in the RSV F adj trial). In low-income countries, overall hospitalization rates may be lower or breast-feeding may be more prevalent^35^, possibly allowing for postpartum antibody transfer and playing a role in protection from RSV disease^37,38^. These differences may impact efficacy estimates and, therefore, prediction accuracy.

The reported efficacy of maternal vaccines RSV F adj and RSVpreF was higher for endpoints related to more severe disease. This effect was not sufficiently represented in the dataset used to develop the MBMA model and, therefore, was not part of the model. Instead, efficacy predictions for RSVpreF were between observed efficacy estimates for RSV MALRI and RSV hospitalization, as anticipated given that the model averaged the efficacies for the different disease severity endpoints. Overall, the model qualification supported the general applicability of the MBMA model for maternal vaccination.

### RSV efficacy simulations for the overall population

Efficacy was subsequently simulated for hypothetical maternal vaccines and clesrovimab, along with the risk of RSV disease for maternal vaccination compared with that for passive immunization with clesrovimab. Consistent with typical birth statistics, only ~4% of the simulated populations were preterm neonates (< 35 weeks of gestation); the remaining percentage was full-term infants, in line with other trials^18,39^. Given the low percentage of preterm infants in the simulated population, predicted SNA titers of the overall population are dominated by those of the full-term population; therefore, titers in the overall population closely resemble those of the full-term population.

Simulation results for prevention of RSV disease for an observation period of 3 months after birth were positive for the overall infant population for all interventions considered: in all simulated scenarios, the efficacies of maternal vaccines and clesrovimab were predicted to be at least 72% (for a GMR of 15) and approximately 75%, respectively, for infants born at the start of or during the RSV season. A maternal vaccine-induced 30-fold increase in infant SNA titers at birth was predicted to achieve protection similar to that predicted for clesrovimab over 3 months. However, because of the more rapid decrease in SNA titers relative to clesrovimab, this level of protection was not maintained for an observation period of 6 months.

Over a 6-month period, a substantial decrease in efficacy was predicted for maternal vaccination with a *GMR*_*infant*_ of 15, whereas one with a greater *GMR*_*infant*_ and clesrovimab maintained efficacy comparable to that of the 3-month observation period. This is consistent with the reported decrease in efficacy for RSVpreF (*GMR*_*infant*_ of 14.25)^19^. Six-month efficacies >75% were maintained only by passive immunization with clesrovimab or by simulated maternal vaccination inducing the highest SNA titer increase (60-fold). Therefore, to provide protection matching what is predicted for clesrovimab, a maternal vaccine would need to increase titers by more than has been reported to date^18^. This is especially the case if the full duration of the RSV season (6 months) is considered.

In addition to efficacy simulations performed in the overall population, Fig. 2 shows that, for a *GMR*_*infant*_ of 15 and a *GMR*_*infant*_ of 60, titers have decreased to levels naturally observed at 3 to 4 months and 5 to 6 months after birth, respectively. Therefore, as also suggested by the RRs (Fig. 4), maternal vaccination is less likely to protect infants from RSV disease if they are born a few months before the RSV season^34^.

### RSV efficacy simulations for subpopulations

While preterm neonates are one of the populations most vulnerable to RSV disease, typical birth statistics indicate that preterm neonates are a small proportion of the overall population. Therefore, it is not likely that ongoing phase 3 trials are powered to define the GA threshold at which it can be assumed that infants have received sufficient transplacental antibody transfer, and, thus, are protected from RSV^40^. To better understand the preventative efficacy of maternal vaccination and clesrovimab in preterm infants, simulation results were stratified into preterm and full-term subpopulations. Efficacy simulation results indicated that protection from RSV disease was nominally greater in preterm neonates than in the full-term population (Table 3): incomplete antibody transfer from the pregnant people^30,31^ to preterm infants results in lower baseline titers at birth and, consequently, a higher placebo RSV incidence rate compared with full-term infants. Irrespective of observation period, these lower titers in preterm infants increase the relative effect size despite similar incidence rates in active (non–placebo control) arms of full-term and preterm infants (data not shown).

Thus, the efficacy depends on population. Based on our simulation results, passive immunization could provide even more substantial and necessary protection in two populations: preterm infants and infants born outside of the RSV season. In preterm infants, results for clesrovimab show that efficacy for this antibody is expected to be similar for 3- and 6-month periods, and efficacy may be even higher in the more vulnerable preterm population than for full-term infants. For infants born outside of the RSV season, passive immunization can be timed relative to the start of the season. This is not feasible for maternal vaccination. Time point selection for maternal vaccination may even prove difficult for infants born shortly before the anticipated RSV season because exact timing of an upcoming RSV season (or of delivery) is unknown.

RSV efficacy simulations for maternal vaccination show that preterm infants born to pregnant people who were previously vaccinated with RSVpreF^19^ will not be well protected over the full RSV season. A 15-fold increase in titers yields a predicted 6-month efficacy of only 46.9% in the preterm population, considerably less than the 62.2% predicted for full-term infants. This reduced RSV efficacy in preterm relative to full-term infants is in line with previously published simulations for these subgroups^17^. The lower efficacy of maternal vaccination for the preterm population is expected given the substantially lower simulated (and expected) SNA titers in preterm infants compared with full-term infants^30,31^, and the decrease within 3 months after birth (Fig. 2) toward levels observed in infants born to unvaccinated pregnant people.

This analysis is based on the fundamental assumption that efficacy is sufficiently determined by SNA titer at the time of RSV exposure. While SNA titer has been demonstrated to be a strong correlate of protection^9^, other factors such as breastfeeding status, size of household, location, and birthdate may also impact an individual’s risk for RSV infection^41–43^. As has been discussed previously^9^, there are also limitations relating to challenges in appropriately using heterogeneous data across trials to build the MBMA model.

Across all simulations performed (i.e., model qualification, simulations for overall population, and for population strata), identical efficacy is predicted for every level of RSV disease severity; this is because of the structure of the MBMA model. Maternal vaccination literature indicates that it may be easier to protect from more-severe RSV disease^18,19^. During MBMA model development, underlying data did not support this differentiation between endpoints, and such trends may not be generalizable for newborns^12^. Nevertheless, point estimates may need to be interpreted more qualitatively (e.g., low, intermediate, or high efficacy). Despite this limitation, the model qualification is sufficiently robust to estimate RR, for example, between different maternal vaccination ratios or different types of interventions, and the results and conclusions hold even if such estimation is not perfect.

In conclusion, a model that predicts RSV maternal vaccination efficacy in neonates was successfully implemented and qualified using reported efficacy results, suggesting that the model framework could reliably predict relative risk between neonate populations for maternal vaccination and those passively immunized using clesrovimab or alternative passive prophylactic approaches.

To provide 6-month efficacy comparable to that predicted for passive immunization with clesrovimab, simulations indicate that a maternal vaccine would need to increase titers by 60-fold, higher than observed increases reported to date. Simulation results also suggest that passive immunization with clesrovimab could provide substantially more protection for preterm neonates and for infants born outside of the RSV season than maternal vaccination (with induced titers reported to date). Model simulations show that, if infants are born just before or during the RSV season, maternal vaccination may provide partial protection from RSV disease for full-term infants. Furthermore, this work suggests that it could be useful to leverage this paradigm for similar models of other pathogens as seen, for example, in the work of Kandala et al.^44^.

## Supplementary information

Supplementary Data 1

Supplementary Data 2

Supplementary Data 3

Reporting Summary

Supplementary Material

## Data Availability

Merck Sharp & Dohme LLC, a subsidiary of Merck & Co., Inc., Rahway, NJ, USA (MSD) is committed to providing qualified scientific researchers access to anonymized data and clinical study reports from the company’s clinical trials for the purpose of conducting legitimate scientific research. MSD is also obligated to protect the rights and privacy of trial participants and, as such, has a procedure in place for evaluating and fulfilling requests for sharing company clinical trial data with qualified external scientific researchers. The MSD data sharing website (available at: https://trialstransparency.msdclinicaltrials.com/policies-perspectives.aspx) outlines the process and requirements for submitting a data request. The summary-level data used in this study is available in the publications that have been referenced herein. The numerical data used to plot Figs. 2, 3, and 4 (source data) can be found in Supplementary Data 1, 2, and 3, respectively.
