# Supplementary material for: Efficacy of monoclonal antibodies and maternal vaccination for prophylaxis of respiratory syncytial virus disease
Source: Commun Med (Lond). 2025 Apr 16;5:119. doi: 10.1038/s43856-025-00807-9 (PMC12003833; doi:10.1038/s43856-025-00807-9)
Supplement: Supplementary file 5 — Supplementary Material [file 43856_2025_807_MOESM5_ESM.docx]

**Supplementary Material**

**Efficacy of monoclonal antibodies and maternal vaccination for prophylaxis of respiratory syncytial virus disease**

Nele Plock^1^, Jeffrey R. Sachs^2^, Xiaowei Zang^2^, Jos Lommerse^1,†^, Kalpit A. Vora^2^, Andrew W. Lee^2, ‡^, S. Y. Amy Cheung^1^, Brian M. Maas^2^

^1^Certara, Radnor, PA, USA; ^2^Merck & Co., Inc., Rahway, NJ, USA

^†^Current affiliation: Nalma, Oss, The Netherlands

^‡^Current affiliation: Uniquity Bio, Malvern, PA, USA

**Supplementary Methods**

**Derivation of serum neutralizing antibody titers for infants born to vaccinated pregnant people**

*Infants_vac_* refers to the population of infants born to pregnant people vaccinated during their pregnancy, and those born to pregnant people who were not vaccinated before delivery as *Infants_nonvac_*. The modeling described here is illustrated in Fig. S1.

A commonly reported value in maternal vaccination studies is the geometric mean ratio (GMR, or fold increase) of serum neutralizing antibody (SNA) titers at birth in *Infants_vac_* to SNA titers of infants in *Infants_nonvac_*. SNA titers in *Infants_vac_* for the first 30 days after birth are modeled as:

$${1) SN}_{vac,i}\left( t \right)={SN}_{nonvac,i}(t)\cdot{GMR}_{infant,i} (for 0 \leq t \leq30)$$

Where *SN_vac.i_ (t)* is the simulated individual SNA titer value at time, *t*, for the *i*th infant in *Infants_vac_. SN_nonvac,i_* *(t)* is the simulated individual SNA titer at time, *t*, for the corresponding *i*th infant in *Infants_nonvac_.* This is calculated using an empirical model (sum of two exponentials) for longitudinal endogenous SNA titer developed previously for full-term and preterm infants^1^:

$$2) log2 SNA=A\cdot e^{\frac{-age}{\tau}} + A\cdot\left( 1-e^{\left( - \frac{\left( age-b \right)}{\tau} \right)\cdot c} \right)$$

where age is the infant’s postnatal age in weeks. This empirical model has been described in more detail in the supplementary material of a previous publication^1^ (including Fig. S9 of that publication, which visualizes model fit to data for several components of this model).

*GMR_infant,i_* is the geometric mean ratio of SNA titer at birth for the *i*th infant of *Infants_vac_* to the *i*th infant of *Infants_nonvac_*, where the ratio for each such pair of infants is sampled from GMR distributions for the respective maternal vaccine

Newborns do not develop their own SNAs during their first weeks of life. However, because of placental transfer of maternal immunoglobulin, infants are born with endogenous respiratory syncytial virus (RSV) neutralization activity that decreases over time as the infant’s circulating (placentally transferred, maternal) antibodies are naturally cleared. An apparent increase in the average half-life of SN titers in infants aged 6 months and older has been linked to boosts in titers following natural RSV infections^2^. However, for infants of vaccinated pregnant people, initial protection from these infections implies that titers averaged across the population would receive a boost only in proportion to the fraction infected. This would be a very small fraction for an efficacious prophylaxis, and the standard initial half-life of 30 days would still apply. This effectively assumes that the maternal vaccination is (nearly) 100% efficacious during this period: the more efficacious the prophylaxis, the smaller the fraction of infants with boosted titers, and complete protection means that the population average titer will continue to decay with the typical half-life. Therefore, it was assumed that a half-life (*T_1/2_*) estimated from SNA titers during the first 30 days after birth for infants in *Infants_nonvac_,* would also apply to SNA titers of infants in *Infants_vac_* from day 30 day until a time *T_delay,i_* defined in the equation that follows. The SNA titers for infants in *Infants_vac_* at time *t* would now be for *t* between 30 days and *T_delay,i_*

$$3) {SN}_{vac,i}\left( t \right)={SN}_{vac,i}(30)\cdot2^{-(t-30)/T_{½,i}} ( for 30 \leq t \leq T_{delay,i})$$

where

- *SN_vac,i_* (30) is the simulated individual SNA value 30 days after birth for the *i*th infant in *Infants_vac_*
- *T_1/2,i_* is the half-life for the *i*th infant in *Infants_vac_* obtained from fitting the SNA titer values of the *i*th infant between times *t* = 0 and *t* = 30 days
- *T_delay,i_* is the time at which *SN_vac,i_* (*t*) has decreased to the titer at birth of the corresponding infant in *Infants_nonvac_* so that *SN_vac,i_* (*T_delay,i_*) = *SN_nonvac,i_* (0)

After *T_delay,i_*, as antibody levels fall below the baseline of their virtual twin in the control group, infants born to vaccinated pregnant people were assumed to experience a similar trajectory as those of their virtual twin after birth (i.e. delay of half-life increase due to natural boost of titers). Starting at *T_delay,i_*, the titer versus time profile for the *i*th infant in *Infants_vac_,* *SN_vac,i_* (*t*), was therefore assumed to be identical to that of the virtual twin infant in *Infants_nonvac_* starting at birth, so that for *t ≥ T_delay_*,

4) ${SN}_{vac,i}\left( t \right)= {SN}_{nonvac,i}\left( t-T_{delay,i} \right) (for T_{delay,i} \leq t )$

This assumption of delayed natural titer after maternal vaccination is supported by findings from previous investigations that show that maternally derived antibodies are the most important factor influencing SNA response to natural RSV infection for the first few months of life.

This model was able to predict an infant’s SNA titer time course from birth onward as a function of an infant’s gestational age.

**Supplementary Figures**

**Fig. S1. Illustration of simulation approach to derive SNA titers for infants born to vaccinated pregnant people.** GMR, geometric mean ratio; SN, serum neutralizing.

**
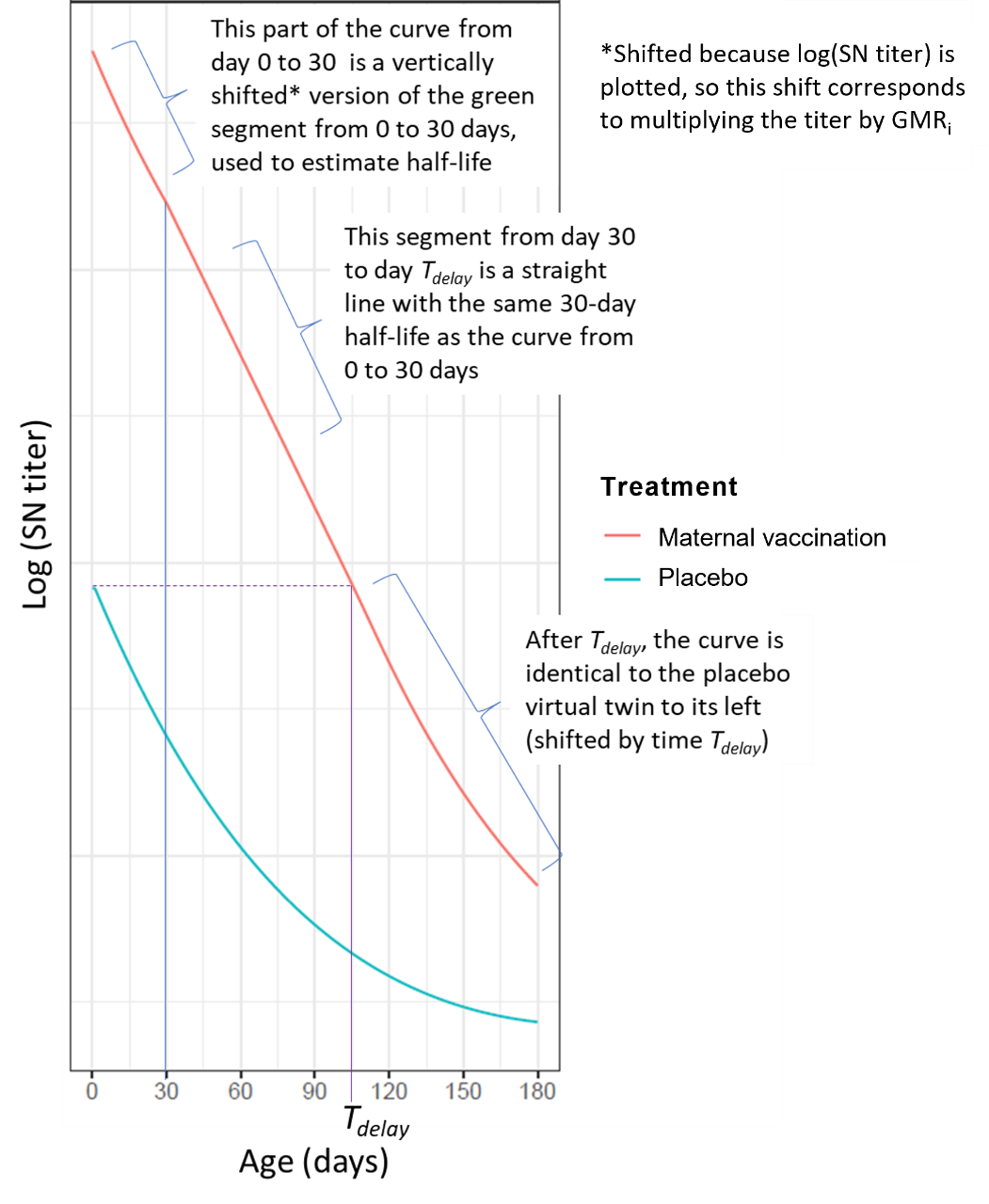
**

**Fig. S2. Clesrovimab and maternal vaccine partial and full RSV season efficacy.** The top and bottom rows show predicted results for observation periods of 3 and 6 months, respectively. Data are presented as mean predicted efficacy with associated 95% confidence interval. Simulated efficacies apply for all RSV end points: data available (for the MBMA model) did not enable different predictions for different RSV end points; therefore, only a single prediction is available. As explained in the main manuscript, overlap in the confidence intervals does not necessarily imply comparable protection. GMR, geometric mean ratio; MBMA, model-based meta-analysis; RSV, respiratory syncytial virus.


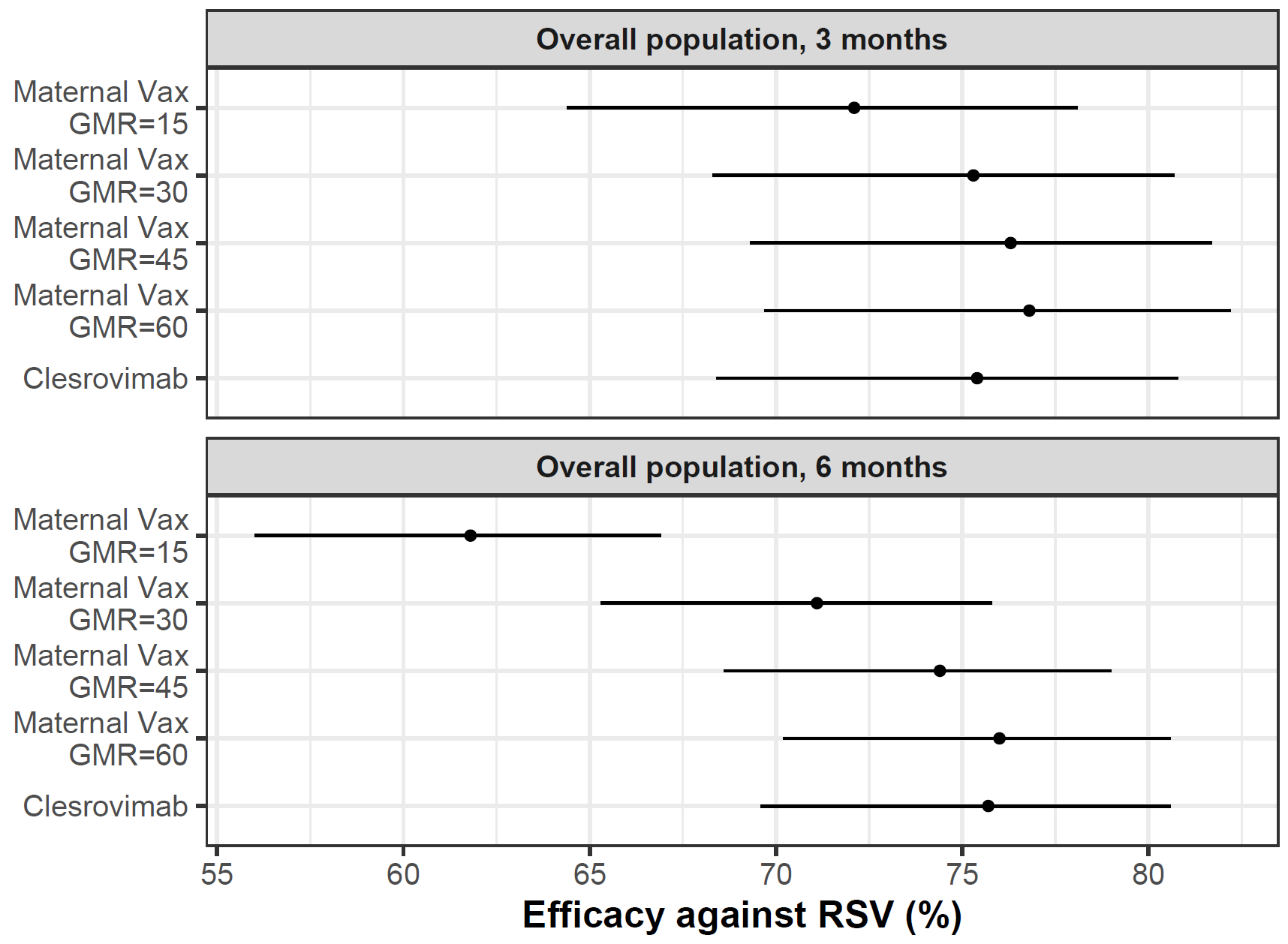


**Fig. S3. Efficacy against RSV by gestational age strata: clesrovimab efficacy is predicted to be more durable than that of hypothetical maternal vaccines.** The top and bottom rows show results for observation periods of 3 and 6 months, respectively. Data are presented as mean predicted efficacy with associated 95% confidence interval. Simulated efficacies apply for all RSV end points: data available (for the MBMA model) did not enable different predictions for different RSV end points; therefore, only a single prediction is available. As explained in the main manuscript, overlap in the confidence intervals does not necessarily imply comparable protection. GMR, geometric mean ratio; MBMA, model-based meta-analysis; RSV, respiratory syncytial virus; wGA, weeks of gestational age.


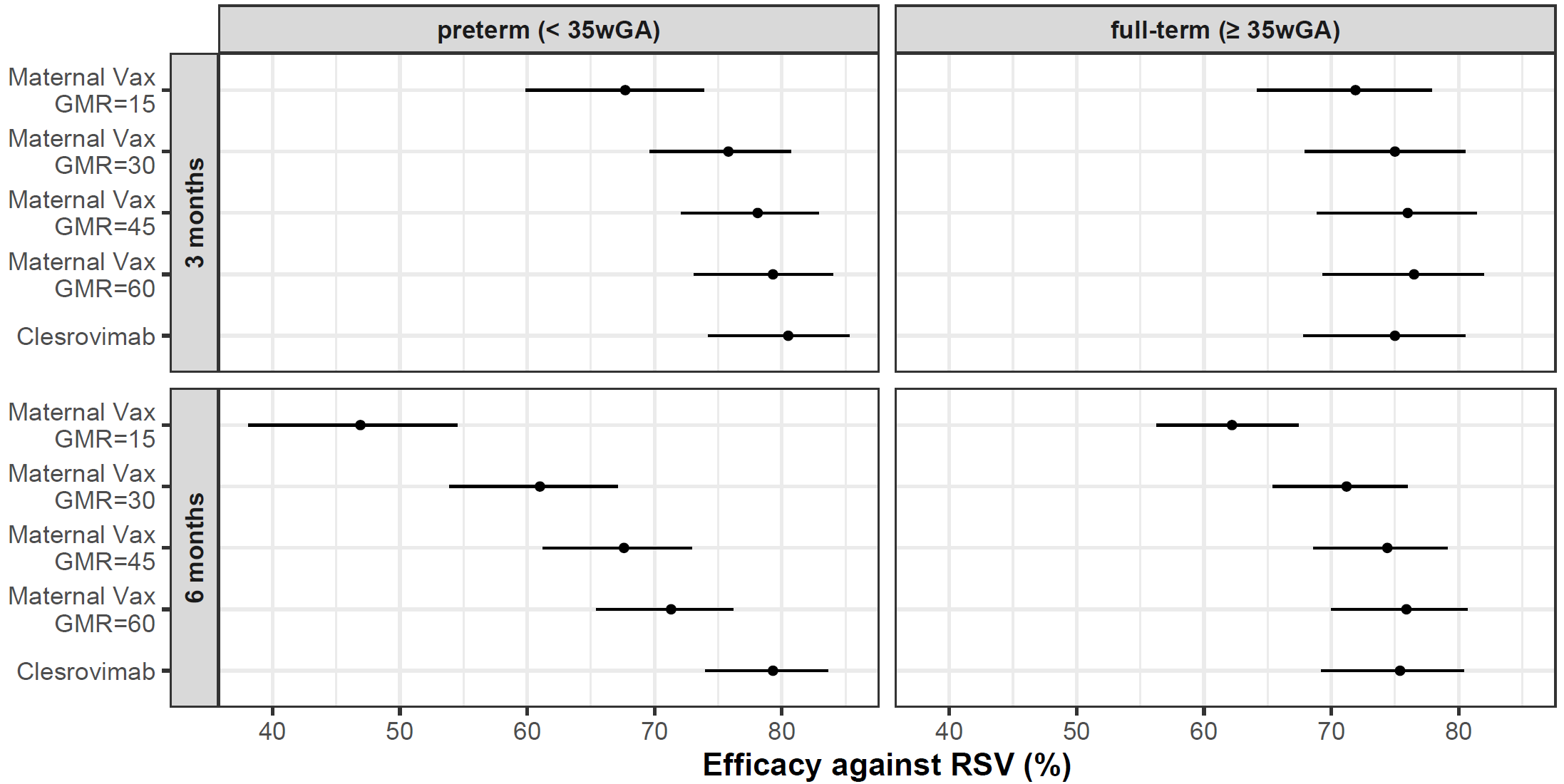


**Table S1.** Model qualification: predicted maternal vaccination RSV efficacies are consistent with reported values.

| Vaccine | Observation period | Predicted efficacy,^a^  % [95% CI] | Observed MALRI efficacy % [95% CI] | Observed hospitalization efficacy % [95% CI] |
| --- | --- | --- | --- | --- |
| RSV F adj | 3 months | 27.3 [21.6-32.5] | 39.4 [5.3-61.2] | 44.4 [19.6-61.5] |
| RSVpreF | 3 months | 71.7 [63.8-77.8] | 57.1 [14.7-79.8] | 81.8 [40.6-96.3] |
| RSVpreF | 6 months | 60.9 [54.9-66.1] | 51.3[29.4-66.8] | 69.4 [44.3-84.1] |

Observed values taken from Madhi et al.^3^ and Kampmann et al^4^. For RSV F adj, primary end point “RSV-associated medically significant lower respiratory tract infection up to 90 days of life” and “hospitalization for RSV-associated lower respiratory tract infection up to 90 days of life” were used to compare against simulated RSV MALRI and RSV hospitalization efficacies, respectively. For RSVpreF, the end points were “medically attended lower respiratory tract illness” and “severe medically attended lower respiratory tract illness,” respectively, with the assumption that severe illness would correspond to hospitalization.
^a^Applies to both RSV MALRI and RSV hospitalization (see Fig. 1, main manuscript). MALRI, medically attended lower respiratory tract illness; RSV, respiratory syncytial virus; RSV F adj, adjuvanted RSV fusion protein (F) vaccine; RSVpreF, RSV F vaccine with prefusion conformation.

**References**

1 Maas, B. M. *et al.* Forward and reverse translational approaches to predict efficacy of neutralizing respiratory syncytial virus (RSV) antibody prophylaxis. *EBioMedicine* **73**, 103651 (2021).

2 Shinoff, J. J. *et al.* Young infants can develop protective levels of neutralizing antibody after infection with respiratory syncytial virus. *J Infect Dis* **198**, 1007-1015 (2008).

3 Madhi, S. A. *et al.* Respiratory Syncytial Virus Vaccination during Pregnancy and Effects in Infants. *N Engl J Med* **383**, 426-439 (2020).

4 Kampmann, B. *et al.* Bivalent Prefusion F Vaccine in Pregnancy to Prevent RSV Illness in Infants. *N Engl J Med* **388**, 1451-1464 (2023).
